# EARLY LIFE ADVERSITY, ALLOSTATIC LOAD, AND COGNITIVE FUNCTION AMONG MIDDLE-AGED AND OLDER ADULTS

**DOI:** 10.1093/geroni/igac059.2255

**Published:** 2022-12-20

**Authors:** Danielle D'Amico, Maya Amestoy, Alexandra Fiocco

**Affiliations:** Toronto Metropolitan University, York, Ontario, Canada; University of Toronto Scarborough, Thornhill, Ontario, Canada; Toronto Metropolitan University, Toronto, Ontario, Canada

## Abstract

Early life adversity (ELA) is consequential for poor cognitive health in mid to late life. ELA is associated with higher allostatic load (AL), a biological indicator of physiological dysregulation due to cumulative wear-and-tear from chronic stress. Higher AL is also associated with poorer cognitive function across the lifespan. To date, however, a paucity of research has investigated AL as a mechanism through which ELA impacts cognition. Using cross-sectional data from the Midlife in the United States (MIDUS) Study, the objective of this study was to investigate the mediating role of AL in the relationship between ELA and cognitive performance (global cognition, episodic memory, executive function) among middle-aged and older adults without cognitive impairment (n=1541, mean age=53±12, 53% female). ELA, including physical, emotional, and sexual experiences, was measured retrospectively using the Childhood Trauma Questionnaire. AL was composed of 20 biomarker proxies of neuroendocrine, metabolic, inflammatory, and cardiovascular systems, stratified by sex. Cognitive performance was evaluated using a battery of neuropsychological tests from the Brief Test of Adult Cognition by Telephone. Controlling for age, education, and ethnicity, AL significantly mediated the relationship between ELA and global cognition (ß=-0.01,95%CI[-0.02,-0.003]) and executive function (ß=-0.01,95%CI[-0.02,-0.003]) such that higher ELA was associated with higher AL, and higher AL was associated with poorer global cognition and executive function. No such effects were found for episodic memory. Consistent with the biopsychosocial lifespan model of cognitive aging, findings suggest that ELA may become biologically embedded over time to negatively impact cognitive function in later adulthood in a domain-specific manner.

